# Rs4862705 in the melatonin receptor 1A gene is associated with renal function decline in type 1 diabetes individuals

**DOI:** 10.3389/fendo.2024.1331012

**Published:** 2024-03-14

**Authors:** Gustavo Daher, Daniele Pereira Santos-Bezerra, Ana Mercedes Cavaleiro, Tatiana Souza Pelaes, Sharon Nina Admoni, Ricardo Vessoni Perez, Cleide Guimarães Machado, Fernanda Gaspar do Amaral, José Cipolla-Neto, Maria Lúcia Correa-Giannella

**Affiliations:** ^1^ Laboratório de Carboidratos e Radioimunoensaios (LIM-18), Hospital das Clínicas HCFMUSP, Faculdade de Medicina, Universidade de São Paulo, São Paulo, Brazil; ^2^ Department of Physiology and Biophysics, Institute of Biomedical Sciences, University of São Paulo, São Paulo, Brazil; ^3^ Divisão de Oftalmologia, Hospital das Clínicas HCFMUSP, Faculdade de Medicina, Universidade de São Paulo, São Paulo, Brazil; ^4^ Department of Physiology, Federal University of São Paulo (UNIFESP), São Paulo, Brazil

**Keywords:** type 1 diabetes mellitus, diabetes-related complications, diabetic kidney disease, antioxidant activity, melatonin receptors, genetic polymorphism

## Abstract

**Aim:**

The pathogenesis of chronic diabetes complications has oxidative stress as one of the major elements, and single-nucleotide polymorphisms (SNPs) in genes belonging to antioxidant pathways modulate susceptibility to these complications. Considering that melatonin is a powerful antioxidant compound, our aim was to explore, in a longitudinal cohort study of type 1 diabetes (T1D) individuals, the association of microvascular complications and SNPs in the gene encoding melatonin receptor 1A (*MTNR1A*).

**Methods:**

Eight SNPs in *MTNR1A* were genotyped in 489 T1D individuals. Besides cross-sectional analyses of SNPs with each one of the microvascular complications (distal polyneuropathy, cardiovascular autonomic neuropathy, retinopathy, and diabetic kidney disease), a longitudinal analysis evaluated the associations of SNPs with renal function decline in 411 individuals followed up for a median of 8 years. In a subgroup of participants, the association of complications with urinary 6-sulfatoxymelatonin (aMT6s) concentration was investigated.

**Results:**

The group of individuals with a renal function decline ≥ 5 mL min^−1^ 1.73 m^−2^ year^−1^ presented a higher frequency of the A allele of rs4862705 in comparison with nondecliners, even after adjustment for confounding variables (OR = 1.84, 95% CI = 1.20–2.82; *p* = 0.0046). No other significant associations were found.

**Conclusions:**

This is the first study showing an association between a variant in a gene belonging to the melatonin system and renal function decline in the diabetic setting.

## 1 Introduction

Diabetes mellitus is one of the major causes of renal insufficiency, blindness, and neuropathic dysfunction worldwide (1). These conditions appear as a result of diabetic microangiopathy, whose pathogenesis is related to the activation of four biochemical pathways by hyperglycemia (polyol, hexosamine, protein kinase C, and the formation of advanced glycation end-products) and consequent cellular damage. Although the evolution of these classical microangiopathic complications, namely, diabetes kidney disease (DKD), retinopathy, and peripheral neuropathy, is directly related to hyperglycemia, it is well documented that glycemic control is not their only predictor (2). For that reason, a more precise definition of susceptibility to these conditions is necessary to address adequate treatment goals and medical support for individuals with diabetes.

Numerous single-nucleotide polymorphisms (SNPs) have been studied in the last two decades, investigating predisposition to diabetic complications. SNPs in genes belonging to pro- and antioxidant pathways have been studied (3–6), since oxidative stress is considered the unifying element of the four biochemical pathways triggered by hyperglycemia (2). SNPs can modulate the susceptibility to diseases by influencing the rate of transcription. Such genetic variants are found in expression quantitative trait loci (eQTL), which are genomic regions (spanning one or more genes) that affect the expression of a given gene and, thus, contribute to the phenotypic variation of a biological trait (7).

Melatonin hormone is synthesized mainly in pinealocytes in a biochemical process that requires two crucial enzymes: arylalkylamine *N-*acetyltransferase (AANAT) and acetyl-serotonin-*O*-methyltransferase (ASMT). Melatonin can act either through nonreceptor-mediated mechanisms or by direct interaction with melatonin receptors 1A (MTNR1A) and 1B (MTNR1B), present in most tissues and organs (8).

Once produced by the pineal gland, the hormone is rapidly metabolized by the liver, mainly by the CYP1A2 enzyme, and is subsequently conjugated to form 6-sulfatoxymelatonin (aMT6s), its principal metabolite that is excreted in the urine, where its measurement is vastly adopted as an indirect, however accurate, method to quantify melatonin production (9).

Melatonin circadian production is synchronized to the external photoperiod; its production is tightly linked to the darkest period of the day. Circadian variations in the metabolism were already well described, including in individuals with type 1 diabetes (T1D) (8).

Another important feature of melatonin is its antioxidant capability; melatonin and its metabolites can act both directly, scavenging reactive nitrogen and oxygen species (ROS), and indirectly, controlling the expression of antioxidant enzymes (10) both *in vivo* and *in vitro*. Studies suggest that melatonin-combined actions exert a role in different diabetic complications, including DKD (11, 12).

Based on what is already known about melatonin’s participation in glucose metabolism and considering its antioxidant properties, studies of genetic variants that could interfere with melatonin synthesis or action and diabetes have already been published. Most of them addressed the association with type 2 diabetes (T2D) or gestational diabetes (13) and evaluated the gene encoding MTNR1B, even though MTNR1A has been described as a major mediator of melatonin actions (14).

The main objective of this study was to explore associations between SNPs on the gene encoding MNTR1A and four microangiopathic complications in T1D individuals. The possible association between diabetes complications and genes related to melatonin actions has been little investigated.

## 2 Materials and methods

For this longitudinal cohort study, clinical and biochemical data and blood samples for DNA extraction were collected in a diabetes outpatient clinic at a tertiary university hospital. From April 2004 until December 2016, a convenience sample of 489 T1D individuals was engaged. The criteria for T1D diagnosis were hyperglycemia in the presence of positive autoantibodies (islet cell antibodies, glutamic acid decarboxylase, or tyrosine phosphatase-like insulinoma antigen 2) or previous history of diabetic ketoacidosis and undetectable C-peptide. The inclusion criterion for this study was T1D diagnosis for at least 10 years, and noninclusion was the presence of kidney failure (estimated glomerular filtration rate [eGFR] < 15 mL min^−1^ 1.73 m^−2^ or treatment by dialysis) or kidney transplantation. All individuals were receiving intensive insulin therapy. The study protocol was approved by the local ethics committees of the institution and was conducted in compliance with the Declaration of Helsinki. All participants signed an informed consent form.

### 2.1 Evaluation of diabetic complications

Cardiovascular autonomic neuropathy (CAN), distal polyneuropathy, and diabetic retinopathy (DR) were evaluated as previously described (3, 15). An albumin-to-creatinine ratio > 300 mg g^−1^ and/or an eGFR < 60 mL min^−1^ 1.73 m^−2^, as calculated by the CKD-EPI formula (16), defined the presence of DKD. In the cross-sectional analysis, a total of 485 participants were evaluated for DKD.

DR was classified according to the International classification of DR (17); a TRC-NW8 Non-Mydriatic Retinal Camera (Topcon, Oakland, NJ, USA) was used for the purpose of standardized seven-field retinal color photographs, which were analyzed by a single ophthalmologist. For this analysis, the presence of DR was defined as moderate and severe nonproliferative DR or proliferative DR, while the absence of DR was defined as mild nonproliferative DR or no DR. A total of 395 participants were evaluated in the cross-sectional analysis.

Distal polyneuropathy was evaluated by the sum of the neuropathy symptom score and modified neuropathy disability score (18, 19). Measurement of systolic blood pressure after 3 min of standing, Ewing test, and spectral analysis of the heart rate variability were used for the diagnosis of CAN (20). A total of 289 and 291 participants were evaluated for distal polyneuropathy and for CAN, respectively, in the cross-sectional analyses.

The use of antihypertensive drugs not for renal protection purposes or systolic/diastolic blood pressure > 140/90 mmHg defined the presence of arterial hypertension. The use of statin or LDL ≥ 100 mg dL^−1^ is defined as dyslipidemia.

A longitudinal analysis of the eGFR was conducted from recruitment until December 2016 or until kidney failure was reached. A total of 411 individuals, followed by a median time of 8 years, were included in this analysis. The individuals who lost ≥ 5 mL min^−1^ 1.73 m^−2^ year^−1^ were defined as decliners, while those who lost < 5 mL min^−1^ 1.73 m^−2^ year^−1^ were defined as nondecliners.

### 2.2 SNP selection and genotyping

DNA was extracted from peripheral blood mononuclear cells employing a salting-out procedure (21). Eight tag SNPs were selected using the LD TAG SNP Selection platform (National Institute of Environmental Health Science) to cover > 95% of the genetic variability across the gene; a *r* ^2^ ≥ 0.8 and a minor allele frequency (MAF) > 0.1 were considered. The SNPs were genotyped by real-time polymerase chain reaction (Step One Plus, Applied Biosystems, Foster City, CA, USA) as previously described (22), employing predesigned Human TaqMan Genotyping Assays 40×: C_6148222_10 (rs2165666), C_27901105_10 (rs4862705), C_26399857_10(rs684769), C_27017887_10 (rs117287777), C_11782809_10 (rs6553010), C_11782814_10 (rs1946977), C_11824449_10 (rs7687823), and C_31861431_10 (rs13140012) from Thermo Fisher Scientific (Foster City, CA, USA). The genotyping success rate was 98% to 99%, and 5% of the samples were genotyped twice, with a 100% concordance rate. The distribution of the genotypes was in accordance with Hardy–Weinberg equilibrium for all SNPs.

### 2.3 Urinary aMT6s measurement

A subgroup of 94 T1D individuals without kidney failure or kidney transplantation collected all urine from 7:00 pm until the morning first-void urine; the samples were frozen at −70°C until analysis. The individuals recorded light exposure, use of electronic devices, and total duration of sleep during nighttime.

Urinary aMT6s was measured by an enzyme-linked immunosorbent assay (ELISA) commercially available kit (RE54031, Männedorf, Switzerland: IBL International) according to the manufacturer’s instructions. On the same urine sample, creatinine was also measured by a colorimetric assay kit (Rotkreuz, Switzerland: Roche Cobas-Roche, USA). Results were expressed as nanograms of aMT6s per milligram of creatinine.

### 2.4 Statistical analyses

Continuous variables were expressed as median and interquartile intervals, and differences between groups were evaluated by the Wilcoxon/Mann–Whitney *U*-test. Categorical variables were expressed as a percentage of cases, and differences were evaluated by Pearson’s Chi-square test. Genotype analyses were performed considering the co-dominant model, and the associations with the complications were evaluated by binary logistic regression analyses to compute the odds ratio (OR) with 95% confidence interval (CI). All confounding variables were assessed, and those statistically significant and with clinical relevance were included as covariates in the regressive model, as shown in the respective tables. As eight SNPs were studied, correction for multiple comparisons considered the number of independent tests (Meff) based on the degree of linkage disequilibrium between SNPs. Thus, a *p*-value of ≤ 0.006 was considered significant. The software JMP (SAS Institute, Cary, NC, USA) was used for the analyses.

Haplotype analysis was conducted using the online software SHEsis; haplotypes with a frequency < 0.01 were not considered in the analysis, and the associations were evaluated using Pearson’s Chi-square test; a *p*-value of ≤ 0.05 was considered significant (23).

## 3 Results

### 3.1 Clinical results

The last (most recent) characteristics of the 489 participants are shown in **Table 1**. The cohort was composed of 43% of men and 57% of women with a median (interquartile interval) diabetes duration of 25 (18–31.2) years, median HbA1c of 8.3% (7.4%–9.7%) (67 [57–83] mmol mol^−1^) and median age of 37 (30–45.5) years.

**Table 1:** The last (most recent) characteristics of all participants.

	*N* = 489
Sex (% male/% female)	43/57
Age (years)	37 (30–45.5)
Body mass index (kg m^−2^)	23.8 (21.4–26.4)
Hypertension (%)	44
Dyslipidemia (%)	34
Total cholesterol (mg dL^−1^)	171 (147–196)
LDL cholesterol (mg dL^−1^)	93 (76–118)
HDL cholesterol (mg dL^−1^)	58 (46–69)
Triglycerides (mg dL^−1^)	86 (61–124)
eGFR (mL min^−1^ 1.73 m^−2^)	95 (55–114)
Diabetes characteristics	
Age at diagnosis (years)	11 (7–18)
Diabetes duration (years)	25 (18–31.2)
HbA_1_C (%)	8.3 (7.4–9.7)
HbA_1_C (mmol mol^−1^)	67 (57–83)
Insulin dose/weight (UI kg^−1^)	0.63 (0.47–0.83)
Diabetic kidney disease (%)	31.2
Use of medications	
Angiotensin-converting enzyme inhibitors (%)	26.3
Angiotensin II receptor blockers (%)	11.4
Statin (%)	35.7

Results expressed as median and interquartile interval. HbA_1_C, glycated hemoglobin; eGFR, estimated glomerular filtration rate; HDL, high-density lipoprotein; LDL, low-density lipoprotein.

The last (most recent) characteristics of the subgroup of 94 participants with urinary aMT6s measurement are shown in **Table 2**. This subgroup was composed of 36.1% of men and 63.9% of women with a median age of 37 (30.7–47.5) years, a median HbA1c of 8.1% (7.3%–9.1%) (65 [56–76] mmol mol^−1^), and a median diabetes duration of 24 (18–34) years. The prevalence of DKD in this group was 19.1%. Urinary aMT6s concentrations were not different between participants with and without any of the evaluated chronic complications, even after adjustments for light exposure in the night and for use of electronic devices (data not shown).

**Table 2:** The last (most recent) characteristics of the subgroup of participants with urinary aMT6s measurement.

	*N* = 94
Sex (% male/% female)	36.1/63.9
Age (years)	37 (30.7–47.5)
Body mass index (kg m^−2^)	25.6 (23.1–28.5)
Hypertension (%)	46.8%
Dyslipidemia (%)	52.1%
Diabetes characteristics	
Age at diagnosis (years)	13 (6–18.2)
Diabetes duration (years)	24 (18–34)
HbA_1_C (%)	8.1 (7.3–9.1)
HbA_1_C (mmol mol^−1^)	65 (56–76)
Insulin dose/weight (UI kg^−1^)	0.57 (0.48–0.75)
Diabetic retinopathy (%)	27.9
Distal neuropathy (%)	26.5
Cardiovascular autonomic neuropathy (%)	18.2
Diabetic kidney disease (%)	19.1
Absence of any diabetes complication (%)	46.3
Medication in use	
Beta-blockers (%)	9
Calcium channel blockers (%)	10.6
Diuretics (%)	9.5
Angiotensin-converting enzyme inhibitors (%)	32.9
Angiotensin II receptor blockers (%)	11.7
Statin (%)	51
Selective inhibitors of serotonin reuptake (%)	19.1
Benzodiazepines (%)	5.3

Results expressed as a median and interquartile interval. HbA_1_C, glycated hemoglobin.

### 3.2 Genotype analyses

No associations were observed between the SNPs and distal polyneuropathy, CAN, or DKD in the cross-sectional analyses (data not shown).

As for the DR analysis, the most recent characteristics of the participants stratified according to their DR status are presented in **Table 3**. In the group without DR, a higher frequency of the G allele of rs1946977 was observed in comparison to the group with DR (MAF = 24% *vs*. MAF = 18.4%, respectively; *p* = 0.046). After adjustment for confounding factors in the logistic regression analysis (sex, age, hypertension, dyslipidemia, triglyceride concentration, diabetes duration, use of angiotensin-converting enzyme inhibitors, angiotensin II receptor blockers, and statin), a nominal association was observed: the rare allele G conferred protection against DR (OR = 0.63; 95% CI = 0.41–0.96; *p* = 0.032). The remaining SNPs did not present associations with DR (data not shown).

**Table 3:** The last (most recent) characteristics of individuals with type 1 diabetes stratified according to the status of diabetic retinopathy (DR).

	Absence of DR	Presence of DR	*p*-value
*N*	247	148	
Sex (% male/% female)	38.4/61.6	47.2/52.8	0.08
Age (years)	34 (27–42)	40 (33–49.7)	< 0.001
Body mass index (kg m^−2^)	24 (21.7–26.8)	23.4 (20.8–25.9)	0.03
Hypertension (%)	27.0	72.9	< 0.0001
Dyslipidemia (%)	36.1	45.9	0.05
Total cholesterol (mg dL^−1^)	172 (148–195)	166 (145–196)	0.92
LDL cholesterol (mg dL^−1^)	93 (75–109)	91 (77–112)	0.62
HDL cholesterol (mg dL^−1^)	59 (49–72)	55 (43–66)	0.0095
Triglycerides (mg dL^−1^)	79 (58–112)	86 (65–124)	0.02
eGFR < 60 mL min^−1^ 1.73 m^−2^ (%)	23.7	76.3	< 0.0001
eGFR (mL min^−1^ 1.73 m^−2^)	105 (87–118)	53 (12–96.5)	< 0.0001
Diabetes characteristics			
Age at diagnosis (years)	12 (7–18)	11 (6–16)	0.22
Diabetes duration (years)	21 (16–29)	28 (23–35)	< 0.0001
HbA_1_C (%)	8.2 (7.4–9.3)	8.4 (7.6–9.6)	0.19
HbA_1_C (mmol mol^−1^)	66 (57–78)	68 (60–81)	0.19
Insulin dose/weight (UI kg^−1^)	0.64 (0.48–0.86)	0.62 (0.47–0.84)	0.57
Medications in use			
Angiotensin-converting enzyme inhibitors (%)	25.9	39.1	0.006
Angiotensin II receptor blockers (%)	10.1	17.5	0.03
Statin (%)	36.8	49.3	0.015

Results expressed as median and interquartile interval. HbA_1_C, glycated hemoglobin; eGFR, estimated glomerular filtration rate; HDL, high-density lipoprotein; LDL, low-density lipoprotein.

Baseline characteristics of the 411 participants included in the longitudinal analysis and stratified according to renal function decline status are shown in **Table 4**. Individuals classified as decliners had a lower body mass index (BMI; 22.3 kg m^−2^ *vs*. 23.5 kg m^−2^, respectively), a higher prevalence of hypertension (33.3% *vs*. 20.3%, respectively), dyslipidemia (44.5% *vs*. 19.6%, respectively), and an eGFR < 60 mL min^−1^ 1.73 m^−2^ (15.1% *vs*. 4.4%, respectively) in comparison to nondecliners. They also had higher concentrations of total cholesterol (186 mg dL^−1^ *vs*. 165 mg dL^−1^, respectively), LDL-cholesterol (105 mg dL^−1^ *vs*. 87 mg dL^−1^, respectively), and triglycerides (105 mg dL^−1^ *vs*. 66 mg dL^−1^, respectively).

**Table 4:** The baseline characteristics of individuals with type 1 diabetes included in the longitudinal analysis stratified according to the status of renal function decline.

	Nondecliners	Decliners	*p*-value
*N*	312	99	
Sex (% male/% female)	42.3/57.7	47.4/52.6	0.36
Age (years)	29.5 (22–40)	29 (24–37)	0.95
Body mass index (kg m^−2^)	23.5 (21.8–25.8)	22.3 (20.2–25.3)	0.05
Hypertension (%)	20.3	33.3	0.02
Dyslipidemia (%)	19.6	44.5	< 0.0001
Total cholesterol (mg dL^−1^)	165 (143–188)	186 (158–223)	< 0.001
LDL cholesterol (mg dL^−1^)	87 (69–104)	105 (82–142)	< 0.001
HDL cholesterol (mg dL^−1^)	60 (52–73)	59 (48–73)	0.31
Triglycerides (mg dL^−1^)	66 (50–96)	105 (72–144)	< 0.0001
eGFR < 60 mL min^−1^ 1.73 m^−2^ (%)	4.4	15.1	0.001
eGFR (mL min^−1^ 1.73 m^−2^)	111 (93–125)	105 (77–127)	0.16
Diabetes characteristics			
Age at diagnosis (years)	12 (7–18)	11 (7–16)	0.76
Diabetes duration (years)	17 (10–24)	16 (12–22)	0.59
HbA_1_c (%)	8.4 (7.2–9.9)	8.2 (7.6–10.1)	0.71
HbA_1_c (mmol mol^−1^)	68 (55–85)	66 (60–87)	0.71

Results expressed as median and interquartile interval. HbA1c, glycated hemoglobin; eGFR, estimated glomerular filtration rate; HDL, high-density lipoprotein; LDL, low-density lipoprotein.

The A allele of rs4862705 was observed at a higher frequency in decliners in comparison to nondecliners (*p* = 0.0114). In the logistic regression analysis, after adjustment for confounding variables, this association was confirmed (OR =1.84; 95% CI = 1.20–2.82; *p* = 0.0046). The remaining SNPs did not present associations with renal function decline (**Table 5**).

**Table 5:** The genotype frequencies of individuals with type 1 diabetes included in the longitudinal analysis stratified according to the status of renal function decline.

	Nondecliners	Decliners	OR (95% CI)	*p*-value
***N***	312	99		
*MTNR1A*				
**rs4862705**			1.84 (1.2–2.82)	0.0046
AA	0.077	0.091		
AG	0.330	0.485		
GG	0.593	0.424		
MAF	0.241	0.333		
**rs2165666**			1.24 (0.83–1.85)	0.2789
TT	0.423	0.444		
TC	0.433	0.394		
CC	0.144	0.162		
MAF	0.360	0.338		
**rs11728777**			0.95 (0.63–1.45)	0.8459
AA	0.234	0.232		
AG	0.519	0.556		
GG	0.247	0.212		
MAF	0.493	0.510		
**rs6847693**			1.01 (1.0–1.01)	0.8312
TT	0.032	0.020		
TC	0.276	0.293		
CC	0.692	0.687		
MAF	0.169	0.166		
**rs1946977**			1.18 (0.72–1.90)	0.4945
GG	0.045	0.030		
GT	0.301	0.384		
TT	0.654	0.586		
MAF	0.195	0.222		
**rs6553010**			1.28 (0.48–3.31)	0.6060
AA	0.048	0.053		
AG	0.321	0.330		
GG	0.631	0.617		
MAF	0.208	0.218		
**rs13140012**			0.86 (0.55–1.33)	0.5248
AA	0.131	0.172		
AT	0.529	0.475		
TT	0.340	0.353		
**rs7687823**			0.96 (0.63–1.46)	0.8809
GG	0.186	0.172		
GA	0.500	0.525		
AA	0.314	0.303		
MAF	0.435	0.434		

CI, confidence interval; MAF, minor allele frequency; OR, odds ratio. Adjusted for arterial hypertension, estimated glomerular filtration rate, triglycerides, and LDL-cholesterol concentration at baseline, and use of angiotensin-converting enzyme inhibitors. p ≤ 0.006 was considered significant.

The haplotype analysis revealed that the haplotype CACAGGAA (rs2165666, rs4862705, rs684769, rs117287777, rs6553010, rs1946977, rs7687823, and rs13140012, respectively), which includes the minor allele A of rs4862705, was associated with an increased risk of DKD (OR = 4.187; 95% CI = 1.07–17.2; *p* = 0.031) and of renal function decline (OR = 7.635; 95% CI = 1.39–41.7; *p* = 0.005). Although these associations have reached statistical significance, the number of individuals with this haplotype (seven patients in total) is extremely limited.

## 4 Discussion

The presence of the minor allele A of rs4862705 in the *MTNR1A* gene conferring risk for renal function decline in T1D individuals was the main finding of this study. Although there is evidence linking melatonin to DKD (24), to the best of the authors’ knowledge, this is the first study showing an association between a variant in a gene belonging to the melatonin system and renal function decline in the diabetic setting.

It is well known that melatonin exerts antioxidant and anti-inflammatory effects and modulates the renin-angiotensin system (RAS) and the sympathetic tone (12, 25). Melatonin antioxidant actions are related to pathways, such as sirtuin 1, that are dependable on MTNR1A signaling (26). MTNR1A activation has also been implicated in the anti-apoptotic as well as in part of the anti-inflammatory effects of melatonin (27). Studies on animal models have demonstrated that melatonin effects on the sympathetic tone are also mediated by MTNR1A activation. This receptor is present in vascular tissues, and its activation is associated with vasoconstriction (28).

Considering that oxidative stress, inflammation, and sympathetic activation participate in the etiopathogenesis of DKD (2, 29), it is plausible that variants in the gene encoding MTNR1A modulate the susceptibility to renal function decline in the context of diabetes mellitus.

In an *in vitro* study using mouse podocytes damaged by angiotensin II—similar to what is found in DKD pathogenesis—exposure to melatonin attenuated ROS generation, resulting in less apoptosis and restoration of cell viability, among other protective effects (11). Thus, in theory, a lower renal action of melatonin secondary to genetic variants could increase the susceptibility to podocyte injury by local angiotensin II, which is stimulated by high glucose concentration (30). Additionally, *in vitro*, albumin was able to reduce MTNR1A expression in tubular epithelial cells (31). Since tubular cells are exposed to albumin in individuals with DKD and albuminuria, it is possible that during the progression of this complication, MTNR1A downregulation participates in renal tubular damage by reducing the beneficial effects of melatonin. We speculate that this process could be exacerbated in individuals with genetic variants that modulate *MTNR1A* expression, a hypothesis that requires confirmation. Alternatively, this SNP may affect another gene. According to the GTEx Consortium Atlas, rs4862705 is an eQTL in the kidney cortex; that is, it affects gene expression by modulating the transcription rate of *FAT1*, the gene neighbor to *MTNR1A. FAT1* encodes FAT atypical cadherin 1, a protein expressed in podocytes and renal tubular cells whose mutations cause glomerulo-tubular nephropathy (32).

The haplotype analysis showed that a haplotype that includes the minor allele A of rs4862705 increased the risk for DKD and renal function decline. These results were presented considering the exploratory nature of this study, but they should be interpreted with caution given the limited number of individuals carrying this haplotype.

The presence of the minor allele G of rs1946977 in the *MTNR1A* gene conferred protection against DR, but the *p*-value did not reach the threshold corrected for multiple comparisons (≤ 0.006). We did not find evidence of the involvement of this SNP with other medical conditions, and further studies are necessary to confirm this finding.

In the urinary analysis, we found no associations between aMT6s concentration and any of the chronic diabetes complications. There are scarce studies exploring the association between diabetes complications and melatonin concentration. Other groups reported lower concentrations of melatonin in individuals with DR using different methods, and Tutuncu et al. reported lower serum melatonin concentrations in association with CAN (33, 34). It is important to notice that all these studies were performed in individuals with T2D, and, to our knowledge, this is the first one carried out in T1D individuals.

The main limitations of this study are the absence of validation in an independent cohort of T1D individuals and functional assays to establish the mechanisms by which rs4862705 could affect renal function. Even though this SNP is an eQTL for a gene relevant to the kidney, its causative role cannot be inferred at this moment; quantitative traits are complex and can be controlled by several genes that interact among themselves (35). The main strength is the longitudinal analysis of the renal outcome, which has the advantage of using more data than only baseline data, providing stronger evidence for a causal relationship than a cross-sectional analysis.

A small, double-blind, placebo-controlled study has demonstrated a decrease in microalbuminuria in T2D individuals receiving melatonin and zinc (36). The association of rs4862705 in the gene encoding MTNR1A with renal function decline may revive interest in studies addressing the use of melatonin in DKD.

## Data availability statement

The raw data supporting the conclusions of this article will be made available by the authors without undue reservation.

## Ethics statement

The studies involving humans were approved by Comissão de Ética para Análise de Projetos de Pesquisa - CAPPesq - Hospital das Clinicas da Faculdade de Medicina da Universidade de São Paulo. The studies were conducted in accordance with the local legislation and institutional requirements. The participants provided their written informed consent to participate in this study.

## Author contributions

GD: Conceptualization, Data curation, Formal analysis, Investigation, Methodology, Validation, Writing – original draft, Writing – review & editing. DS: Data curation, Formal analysis, Methodology, Validation, Writing – review & editing. AC: Methodology, Writing – review & editing. TP: Investigation, Writing – review & editing. SA: Investigation, Writing – review & editing. RP: Investigation, Writing – review & editing. CM: Investigation, Writing – review & editing. FA: Conceptualization, Investigation, Methodology, Resources, Validation, Visualization, Writing – review & editing. JC: Conceptualization, Data curation, Formal analysis, Funding acquisition, Methodology, Resources, Writing – review & editing, Writing – original draft. MC: Conceptualization, Data curation, Formal analysis, Funding acquisition, Project administration, Resources, Supervision, Validation, Visualization, Writing – review & editing, Writing – original draft.
